# Modulation of the main porcine enteric neuropeptides by a single low-dose of lipopolysaccharide (LPS) *Salmonella* Enteritidis

**DOI:** 10.1186/s13099-017-0225-6

**Published:** 2017-12-12

**Authors:** Anita Mikołajczyk, Sławomir Gonkowski, Dagmara Złotkowska

**Affiliations:** 1 Department of Public Health, Epidemiology and Microbiology, Faculty of Health Sciences, University of Warmia and Mazury in Olsztyn, ul. Warszawska 30 Str., 10-082 Olsztyn, Poland; 2 Department of Clinical Physiology, Faculty of Veterinary Medicine, University of Warmia and Mazury in Olsztyn, Oczapowskiego 13 Str., 10-718 Olsztyn, Poland; 3 Department of Food Immunology and Microbiology, Institute of Animal Reproduction and Food Research, Polish Academy of Sciences in Olsztyn, Tuwima 10 Str., 10-748 Olsztyn, Poland

**Keywords:** Low-dose lipopolysaccharide, LPS, *Salmonella* Enteritidis, Neuropeptides, Pig, GAL, NPY, SOM, SP, VIP

## Abstract

**Background:**

The present research was conducted to investigate the influence of a low, single dose of LPS, which does not result in any clinical symptoms of intoxication on the expression of selected neuropeptides within the intestines of the domestic pig.

**Methods:**

This experiment was conducted on immature female pigs of the Pitrain × Duroc breed (n = five per group). Seven days after the intravenous injection of 10 mL saline solution for control animals and 5 μg/kg b.w. (in 10 mL saline solution) LPS *Salmonella* Enteritidis for the experimental group, the excised segments of duodenum, jejunum, ileum, ileocecal valve, caecum, descending colon, transverse colon, ascending colon and rectum were prepared to extract the main enteric neuropeptides, including GAL, NPY, SOM, SP, VIP.

**Results:**

The results of this research indicate that single low-dose LPS *S.* Enteritidis produced changes in the content of the selected neuropeptides of the porcine intestine. The most visible changes were observed in the transverse colon, where LPS induced the increase of GAL expression from 19.41 ± 7.121 to 92.92 ± 11.447 ng/g tissue.

**Conclusion:**

The exact functions of the substances studied and mechanisms of responses to LPS action depend on the sections of the intestines. The mechanisms of observed changes are not fully understood, but fluctuations in neuronal active substance levels may be connected with neurodegenerative and/or pro-inflammatory activity of LPS.

## Background

In recent years, a major topic of research interest has been the rise of the impact of the intestinal barrier, pathogenic and non-pathogenic bacteria on etiology and the pathogenesis or clinical course of neurodevelopmental, psychiatric and neurodegenerative diseases, such as depression, Alzheimer’s disease and Parkinson’s disease. In these pathological processes, neuropeptides have been the main subject of interest for neurodegeneration and neuroprotection. It is known that gastrointestinal physiology involves complex interactions between the nerve cells and the other non-neuronal cell types, for example, enteroendocrine cells. Their peptide secretory products play a role in the regulation of the gastrointestinal (GI) tract functions. Neuropeptides may have an influence on the activity of the GI microbiota and its interaction with the gut–brain axis [1–3].

It is also well-known that the digestive system is supplied by nerves derived from various neuronal cells. The most important role in the regulation of gastrointestinal (GI) tract functions is played by the enteric nervous system (ENS), which is located in the wall of esophagus, stomach, as well as small and large intestine [4]. The ENS is built of millions of neuronal cells connected by dense nerve fibers and grouped into ganglionated plexuses [5]. The number of these plexuses and their exact location clearly depend on the animal species and the fragment of the digestive tract. There are two enteric plexuses in rodents. One of them (myenteric plexus—MP) is placed between the longitudinal and circular muscle layers, while the second (submucosal plexus) is located near the lamina propria of mucosal layer [5]. In the small and large intestine of large animals (including the pig), the submucosal plexus is divided into the outer submucous plexus (OSP)—placed on the internal side of the circular muscle layer—and the inner submucous plexus (ISP)—located like the submucosal plexus in rodents [6]. Enteric neurons are very diverse in their morphology, electrophysiological properties, functions and active substances presented within cell bodies [4]. They regulate all roles of the GI tract, including, among others, intestinal motility, mucosal secretion, mesenteric blood flow or absorption of nutrients [4]. Apart from the ENS, the GI is supplied by extrinsic innervation, in which neuronal cells are localized in sympathetic, parasympathetic and sensory ganglia.

Both extrinsic and intrinsic parts of the digestive system innervation can contain a wide range of active substances, which mainly play the role of neuromediators and/or neuromodulators. Apart from classical neurotransmitters, such as acetylcholine and noradrenaline [7], the most important factors taking part in the regulation of digestive functions include vasoactive intestinal polypeptide (VIP), nitric oxide (NO), galanin (GAL), substance P (SP), neuropeptide Y (NPY) and somatostatin (SOM). Some of these substances, besides the nervous system, are also present in enteroendocrine cells of the digestive tract and can play the function of hormones involved in the intestinal regulatory processes.

One of the lesser-known aspects of innervation of the digestive system is the adaptive and/or neuroprotective activity of biologically active substances during various intestinal and extra-intestinal diseases, inflammatory processes and intoxication [8]. Admittedly, it is known that neurons supplying the digestive organs may change their neurochemical coding under the above-mentioned pathological processes and these changes clearly depend on the type of disease [8], although the exact mechanisms of these changes still remain not fully explained.

On the other hand, it is known that *Salmonella* is one of the common foodborne pathogens and the symptom-free carries of *Salmonella* spp. are still a significant public health problem. Lipopolysaccharide (LPS, bacterial endotoxin)—a component of the cellular membrane of Gram-negative bacteria—is a very important pathological factor [9, 10], which has multidirectional negative effects on the living organism. Lipopolysaccharides from distinct pathogens can induce different responses. First of all, LPS stimulates the release of free radicals and activates the immunological system [11, 12], leading to the injury of internal organs, fever, septic shock and often death [9]. Mohammadi et al. [13] observed that endotoxin of gram-negative bacteria is essentially involved in the pathogenesis of critical illness polyneuropathy in septic patients. Niehaus [14] reported that 14 years after a laboratory worker developed Parkinson’s syndrome after accidental exposure to *Salmonella* Minnesota LPS, the lipopolysaccharides had not been detoxified by the body.

Nevertheless, the knowledge concerning the LPS-induced changes in the expression of neuronal active substances within the digestive tract is extremely limited, especially in the case of low doses of bacterial endotoxin.

Therefore, the aim of the present study was to investigate the influence of a “low single dose” of LPS, which does not result in any clinical symptoms of intoxication [15] on expression of selected neuropeptides within the intestines of the domestic pig. The exposure to a low dose *Salmonella* spp. endotoxin with an absence of clinical symptoms of disease can hypothetically take place during, for example, the asymptomatic carrier state of *Salmonella* spp. It should be pointed out that this animal species is increasingly used as an animal model of processes within the human body due to relatively well-known similarities between these two species with respect to physiological, biochemical and immunological properties [16], particularly in terms of the innervation of digestive organs [17]. Using the pig as a biomedical model which is phylogenetically closer to humans than rodents, plays a critical role in understanding the physiological and pathophysiological processes in the human body [18].

## Results

During this investigation, there were no differences in behavior, feeding habits or health status between animals of the control and experimental groups. Moreover, both macroscopic abduction after euthanasia, as well as histopathological examinations of the particular fragments of the gastrointestinal tract of experimental animals (performed in a specialized veterinary laboratory at the Department of Pathological Anatomy, Faculty of Veterinary Medicine, University of Warmia and Mazury, Olsztyn [Poland]) did not find any changes in comparison to control animals.

**Fig. 1:**
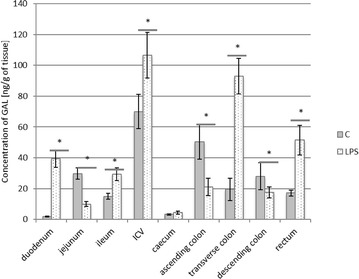
Galanin level in the intestine sections of control (n = 5) and LPS-treated (n = 5) swine. *Statistically different for p < 0.05

In the present study, the expression of all neuropeptides investigated was observed in all examined parts of the digestive system, both in control animals and in pigs after LPS administration. The highest expression was observed for GAL. In the control animals, the level of this substance exceeded 10 (from 14.96 ± 1.953 ng/g tissue in the ileum to 70.02 ± 11.18 ng/g tissue in the ileocecal valve) in almost all fragments of the gastrointestinal tract studied (Fig. 1). The only exceptions were the duodenum and caecum, where the levels of GAL amounted to 1.73 ± 0.261 and 3.21 ± 0.482 ng/g tissue, respectively. LPS administration changes the expression of GAL in all fragments of the digestive tract, except the caecum. The character and intensity of changes clearly depended on the intestinal fragment. In particular, the increase of the GAL expression after LPS administration was observed within the duodenum, ileum, ileocecal valve, transverse colon and rectum, whereas in jejunum and the ascending and descending colon, the levels of this substance were clearly lower (Fig. 1). The most visible changes were observed in the transverse colon, where LPS induced the increase of GAL expression from 19.41 ± 7.121 to 92.92 ± 11.447 ng/g tissue (Fig. 1).

**Fig. 2:**
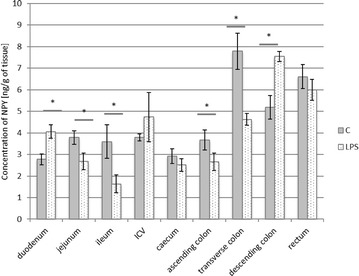
Neuropeptide Y level in the intestine sections of control (n = 5) and LPS-treated (n = 5) swine. *Statistically different for p < 0.05

The second substance, whose level underwent LPS-induced changes in relatively numerous parts of the digestive tract, was NPY. In control animals, the expression of this substance fluctuated from 2.77 ± 0.246 ng/g tissue in the duodenum to 7.79 ± 0.839 ng/g tissue within the transverse colon (Fig. 2). LPS administration caused changes in NPY levels in six investigated fragments of the digestive tract, with their increase within the duodenum and descending colon and a decrease in jejunum, ileum, ascending and transverse colon (Fig. 2). The most visible changes in the expression of NPY after LPS administration were observed in the transverse colon (the decrease from 7.79 ± 0.839 to 4.62 ± 0.272 ng/g tissue) and ileum (the increase was from 3.59 ± 0.78 to 1.63 ± 0.413 ng/g tissue). LPS did not change the NPY levels in the ileocecal valve, caecum or rectum (Fig. 2).

**Fig. 3:**
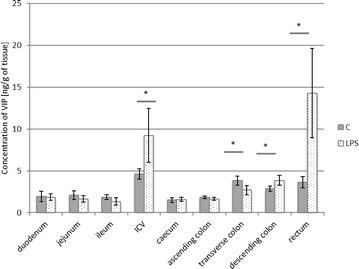
Vasoactive intestinal peptide level in the intestine sections of control (n = 5) and LPS-treated (n = 5) swine. *Statistically different for p < 0.05

In control animals, the levels of VIP fluctuated from 1.50 ± 0.301 ng/g tissue in the caecum to 4.62 ± 0.636 ng/g tissue in the ileocecal valve (Fig. 3). LPS administration caused changes in VIP expression within the ileocecal valve, transverse and descending colon as well as in the rectum. These changes were manifested by a decrease within the transverse colon (from 3.84 ± 0.542 to 2.69 ± 0.551 ng/g tissue) and an increase in the other above-mentioned intestinal parts, with the most visible changes in the rectum (from 3.64 ± 0.671 to 14.29 ± 5.346 ng/g tissue) (Fig. 3).

**Fig. 4:**
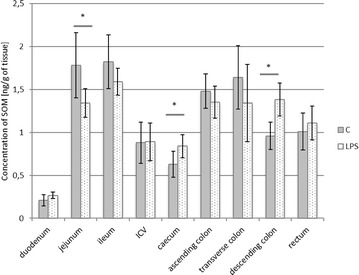
Somatostatin level in the intestine sections of control (n = 5) and LPS-treated (n = 5) swine. *Statistically different for p < 0.05

The levels of SOM observed during the present study in control animals were slightly lower than the expression of VIP and ranged from 0.21 ± 0.066 ng/g tissue in the duodenum to 1.82 ± 0.314 ng/g tissue in the ileum (Fig. 4). LPS administration induced a decrease of SOM levels in the jejunum (from 1.78 ± 0.379 to 1.34 ± 0.167 ng/g tissue), while within caecum and descending colon an increase of SOM expression was observed (from 0.63 ± 0.153 to 0.84 ± 0.134 ng/g tissue and from 0.96 ± 0.157 to 1.38 ± 0.192 ng/g tissue, respectively) (Fig. 4).

**Fig. 5:**
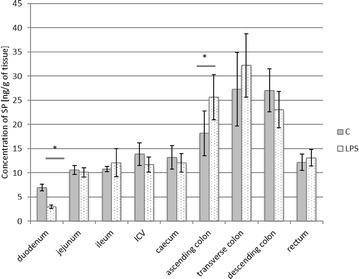
Substance P level in the intestine sections of control (n = 5) and LPS-treated (n = 5) swine. *Statistically different for p < 0.05

In turn, the expression of SP was relatively high in all investigated parts of the digestive tract (Fig. 5). In control animals, it fluctuated from 6.95 ± 0.704 ng/g tissue in the duodenum to above 27 ng/g tissue in the transverse and descending colon. Contrary to the other neuropeptides studied, LPS changed SP levels only in two segments of the digestive tract. An increase in the expression of SP was observed in the ascending colon (from 18.17 ± 4.616 to 25.62 ± 4.71 ng/g tissue), while within the duodenum a decrease in SP levels was noted (from 6.95 ± 0.704 to 2.98 ± 0.355 ng/g tissue).

## Discussion

The obtained results show the presence of all neuropeptides studied in all investigated parts of the digestive system and, hence, confirm the participation of these factors in regulatory processes during digestion and absorption of nutrients, which is in agreement with previous studies [4, 5].

Moreover, it is known that the exact roles of substances studied in the present investigation may depend on the fragment of the intestine [4]. First of all, such a situation has been observed in the case of GAL. This substance takes part in the regulation of gut motility, secretion and other neurotransmitters released by the influence on voltage-dependent Ca^2+^ channels [19–21]. The exact effects of GAL on the alimentary system are different in various animal species. For example, GAL induces smooth muscle contraction in the ileum of the rat, guinea-pig, rabbit and pig [22], while in the canine pylorus and ileum it exhibits relaxatory effects [19]. The results obtained during the present study and significant differences in GAL expression in particular, even contiguous segments of intestine, seems to support the multidirectional activity of this substance. Multidirectional influence on intestinal muscles has been also described in studies on SP, which, acting on the NK3 receptor, causes their contraction and their connection with NK1 receptor shows relaxatory effects [23]. Moreover, SP is considered to be one of the most important neurotransmitters involved in sensory and pain conduction [24], as well as the regulator of blood flow and secretory activity of the digestive system [23]. Other substances studied during the present experiment (NPY, SOM, VIP) have been described within the digestive system as inhibitory factors, which may influence both intestinal motility and secretion [25–27], as well as on mesenteric blood flow [28]. The significant differences in the expression of neuropeptides in particular parts of the digestive system in control animals, as well as various character of changes after LPS administration observed during the present study are probably connected with different exact roles of these substances depending on the fragment of the GI tract.

Moreover, the results of the present study show that LPS may be one of factors affecting the expression of neuropeptides within the digestive system. Previous studies described a wide range of pathological processes and toxic chemical compounds have such properties. The most important of them are inflammatory processes [8], nerve fiber damage [16], mycotoxins [29, 30], extra-intestinal metabolic diseases [31] and psychiatric and neurodegenerative diseases, such as depression or Parkinson’s disease (PD) [32, 33].

Maes et al. [32] confirmed an increased gastrointestinal permeability with a translocation of lipopolysaccharide from gram-negative bacteria may induce depressive symptoms. An understanding of the fact that enterobacteria have antigenic sites which are very similar to those of the lipid structures of neuronal tissue may be crucial for the identification of risk factors of major depressive disorder. The influence of toxins such as MPTP on neurons in the ENS has been associated with the development of PD symptoms. According to Anderson et al. [34], the MPTP PD model was a selective dopaminergic neurotoxin in the ENS and caused changes in colonic motility. In addition to the MPTP PD model, several animal models, for example, the lipopolysaccharide PD model or the rotenone PD model, can reproduce aspects of PD pathology [35, 36]. The enteric nervous system, with their neurons and enteric glial cells which are involved in the regulation of neurotransmission, could be critical in the pathophysiology of Parkinson’s disease [37, 38]. The results of this research indicate that LPS *S.* Enteritidis involved changes in the content of the main neuropeptides (GAL, NPY, SOM, SP, VIP) of the porcine enteric nervous system (Figs. 1, 2, 3, 4, 5). But it is also known that not only pathological agents, but also physiological factors, including the growth and development of the living organism or the diet modification may change the ENS [39].

It should be pointed out that the mechanism of these neuropeptides changes still remains unclear. Fluctuations in the expression of neuropeptides are intended for the protection of secretory cells and adaption of the nervous system to functioning in amended (pathological) conditions [8, 40]. Moreover, a low dose of LPS is known as one of the factors that may change the number and chemical coding of enteric neurons, but knowledge of this subject is extremely limited. In particular, it is known that a single low dose of LPS may cause an increase in the number of intramural gallbladder nerves immunoreactive to a wide range of neuronal factors, including, among others, SP, NPY and GAL, but the mechanisms of these processes are completely unknown [41, 42]. On the other hand, the neuroprotective properties of the majority of substances studied during the present experiment have been reported in previous investigations [4, 8]. Moreover, it is known that they also may affect the inflammatory processes. For example, VIP inhibits the activity of macrophages [43] and SP stimulates the synthesis of interleukins and tumor necrosis factor [23]. In turn, SOM takes part in various pathological processes within the digestive system [44], reduces pain sensation during intestinal inflammation, regulates the immunological response and may act as an anti-inflammatory factor [45].

Previous studies have also described the functions of substances studied in the present investigation during LPS intoxication. It should be pointed out that the knowledge concerning the influence of LPS on the expression of neuropeptides investigated during the present study is very scanty and not limited to experiments on low single doses of bacterial endotoxin. Most of the information concerns the roles of VIP in the immune maintenance during LPS intoxication [46]. It is known that VIP may affect the monocytes stimulated by LPS, and this activity manifests itself in an increase of IL-10, IL-6 and TNFα synthesis [47], which can suggest the possibility of using VIP in adjunctive therapy to antibiotic treatments [48, 49]. On the other hand, some studies have reported that a deficiency of VIP may support resistance to lipopolysaccharide-induced endotoxemia [50]. The results of this research indicate that LPS administration caused a decrease in VIP expression in the transverse colon. Similarly, a decrease in the number of enteric neurons of submucosal plexuses containing VIP was detected by colonoscopy in parkinsonian patients with chronic constipation [33]. Moreover, VIP is a factor that participates in the rescue of enteric neurons from LPS-induced cell death [51]. In the present research, an increase in VIP expression was observed in the ileocecal valve and the transverse and descending colon and in the rectum. Similar neuroprotective functions under LPS action are characteristic of SOM [52], which can also modulate the production of cytokines and chemokines by monocytes treated with bacterial endotoxins [53]. In turn, galanin mRNAs synthesis in the lateral hypothalamic area is reduced under bacterial endotoxin administration [54], and GAL (like NPY and SP) stabilizes body temperature and shows antipyretic effects in an experimental LPS-induced pyrogenic reaction [55–57]. Moreover, NPY modulates immunoreactive reactions after LPS administration [58], improves blood pressure and staves off hypotension during endotoxic shock [59].

Above all, the fluctuations in the expression of neuropeptides noted during the present investigation may be connected with the relatively well-known inflammatory activity of LPS. This activity is connected with the influence of lipid A (one of the components of LPS on macrophages, monocytes and neutrophils), which results in an increase in pro-inflammatory factor synthesis, especially TNF-α [9, 11]. Such supposition is in agreement with previous studies, where the participation of various neuronal factors in processes connected with inflammatory-induced immunological response has been described [8]. On the other hand, low doses of LPS (which did not result in inflammatory processes) were used in the present experiment. However, it seems likely that subclinical inflammatory activity takes place even in the case of low LPS doses.

The second reason for the observed changes may be neuroprotective and/or adaptive processes that respond to LPS-induced disturbances in homeostasis. It is more likely that all neuropeptides studied have been previously described as substances taking part in the protection of the nervous system against different damaging factors [4, 5, 8]. At the same time, LPS is a relatively well-known factor, which may be involved in neurodegenerative processes within the central and peripheral nervous system [60, 61] and these effects are so intense that LPS is one of the substances used to chemically-induce Parkinson’s disease in animal models [62]. Thus, the changes observed during the present study may help protect the intestinal neuronal structures from the neurodegenerative activity of LPS, particularly through the participation of nerves supplying the gastrointestinal tract in reduction of LPS-induced pathological changes as described in previous studies [63].

It cannot be excluded that the observed changes are connected with other direct influences on the nervous system, including the blockade of synaptic transmissions and/or the influence on sensory stimuli conduction [64, 65]. Moreover, the mechanism of fluctuations in the expression of biologically-active substances is also unclear. It may be connected with changes during transcription, translation, post-translational modification of peptides and/or alterations (disturbances or augmentation) of axonal transport, especially from neuronal cell bodies located outside the digestive organs to nerves supplying them.

## Conclusion

To sum up, the obtained results clearly show that even low doses of LPS, which simulate the asymptomatic carrier state of *Salmonella* spp., may affect the expression of neuropeptides in the digestive system. Moreover, significant differences in the intensity and character of observed changes between particular parts of the digestive system strongly suggest that the exact functions of the substances studied and mechanisms of responses to LPS action depend on the fragment of the gastrointestinal tract. The observed changes are probably connected with the neurodegenerative activity of LPS. On the other hand, due to the multidirectional influence of LPS on the living organism, as well as the diverse functions of particular neuropeptides, the full elucidation of the investigated fluctuations is very difficult and requires further studies.

## Methods

### Animals and experimental procedures

This experiment was conducted on 10 immature female pigs of the Pitrain × Duroc breed. The pigs were maintained in individual pens with an area of about 4 m^2^ with a 12-h-long light cycle for 2 weeks prior to the experiment in order to allow adaptation to the new environment. The experiments were conducted when the pigs were 8–9 weeks of age with body weights of 16–18 kg. All animals included in the experiment were clinically healthy and were not carriers of *Salmonella* spp., which was excluded by standard fecal analysis tests. Pigs during experiment were nourished with feed appropriate for pigs of this age group (twice a day in the quantity recommended by producer) and had an access to water ad libitum. All procedures during this investigation were performed according to the recommendations of the Local Ethical Committee for Animal Experimentation in Olsztyn (Poland) (Decision no. 73/2015 from 29th Sept 2015).

After a 2-week adaptive period, pigs included in the experiment were randomly divided into two groups (five pigs in each group): control group (C group) and experimental (LPS group). Animals of both these groups were subjected to premedication, according to the method previously described by Mikolajczyk [66] with intramuscular injection of atropine (Atropinum Sulfuricum Polfa Warszawa S.A., Poland, 0.035 mg/kg b.w.), ketamine (Bioketan, Vetoquinol Biowet Sp. z o.o., Poland and Vetoquinol S.A., France, 7.0 mg/kg b.w.) and medetomidine (Cepetor, CP-Pharma Handelsges mbH, Germany, 0.063 mg/kg b.w.). The premedication of animals allowed accurate and safe (for investigators) injections of LPS. Premedication of control animals was performed to maintain the same conditions for all animals.

Under premedication, the control animals were injected with a 10 mL saline solution, while pigs of the experimental group received lipopolysaccharides from *Salmonella enterica* serotype Enteritidis (catalogue no. L7770 Sigma, Aldrich, Germany) at a dose of 5 μg/kg b.w. (in 10 mL saline solution). Such a dose has been previously described as a “low single dose” which does not result in any clinical symptoms of intoxication [15, 67, 68]. Injections in control and experimental animals were performed in the same way, i.e. intravenously into the marginal ear vein. All procedures and drugs were managed and administered by a veterinary doctor (DVM, Ph.D.).

After a 7-day period which has been described as sufficient for the emergence of changes in the nervous system in the previous studies [69, 70] all animals were again premedicated (in the above-described manner) and subjected to general anesthesia using propofol (Scanofol, NORBROOK, Northern Ireland, IRL.PN, 4.5 mg/kg b.w. given intravenously), and then euthanized with pentobarbital (Morbital, Biowet Puławy Sp. z o.o, Poland, 60–70 mg/kg b.w., given intravenously). After euthanasia, the excised segments of duodenum, jejunum, ileum, ileocecal valve (ICV), caecum, descending colon, transverse colon, ascending colon and rectum (the same fragments from all animals) were washed in 0.9% NaCl, and then packed, frozen in liquid nitrogen and stored at − 80 °C until analysis.

### A sample preparation and solid-phase extraction (SPE)

Neuropeptide extracts from tissue were prepared according to the Conlon procedure [71]. Briefly, frozen tissues were weighed and cut into small pieces. 10 mL of hot 1 M acetic acid was added per gram tissue and boiled for 5 min. The samples were then homogenized using Ultra Turax IKA T-25 (Jankel & Kunkel IKA, Germany) at RT for 5 min and centrifuged at 4 °C for 40 min at 4500×*g* (Eppendorf 5804). The supernatant was filtered by syringe filter with a graduated glass fiber pre-filter (Millex-HPF HV Filter, 0.45 µm, PVDF, Milipore). TFA were added to filtrates to obtain a final concentration of 0.1% (vol/vol). In the SPE method, silica-based cartridges (Sep-Pak Short 360 mg C18, Waters) were used according to the producer’s protocol using a Baker Vacuum Manifold SPE-12G (J.T.Baker, Germany). Samples were concentrated on miVac centrifugal vacuum concentrators, model DNA-23050-800 with SpeedTrap (Genevac Limited, UK) for 2 h, then lyophilized using an ALPHA 1-4 LSC freeze dryer (MARTIN CHRIST Gefriertrocknungsanlagen GmbH Germany). Lyophilized samples were stored at − 80 °C until analysis.

The chemicals used for extraction: glacial acetic acid (cat. no. 951503, J.T. Baker), trifluoroacetic acid –TFA (cat. no. 9470, J.T. Baker) and acetonitrile—LC–MS reagent (cat. no. 9821.1000, J.T. Baker) were of high purity grade—HPLC grade.

### Enzyme immunoassay for quantitative determination of neuropeptides in tissue extracts

Phoenix Pharmaceuticals, Inc. tests for Vasoactive Intestinal Peptide (0–25 ng/mL; cat. no. EK-064-16CE), Neuropeptide Y (0–100 ng/mL; cat. no. EK-049-03CE), Somatostatin-28 (0–25 ng/mL; cat. no. EK-060-14CE) were used for VIP NPY and SOM determination, respectively. The standard protocol proposed by the manufacturer for kits in the ranges of 0–25 and 0–100 ng/mL was used. Briefly, 50 μL of standard, sample or positive control, together with 25 μL of primary antibody and 25 μL of biotinylated peptide were put on a plate and incubated for 2 h at room temperature. The plate was then washed four times with a 350 μL of assay buffer and then 100 μL of streptavidin conjugated with HRP was added. After 1 h incubation and a washing step, 100 μL of TMB substrate was added to each well and the plates were incubated again for 1 h et RT. Reaction was terminated with 100 μL/well of 2 N HCl. Absorbance was read at λ = 450 nm on Infinite 200 (Tecan) for each sample.

Peninsula Laboratories International, Inc. tests for Substance P (0–5 ng/mL; cat. no. S-1180), Galanin—GAL (0–10 ng/m; cat. no. S-1210) were used for SP and GAL determination, respectively. The protocol proposed by the manufacturer to increase sensitivity was used. Briefly, 50 μL of standard or sample, together with 25 μL of the primary antibody, were incubated overnight at 4 °C. The plates were then incubated at RT for 1 h and 25 μL of biotinylated tracer was added to the each well and incubated for 2 h at room temperature. The plates were washed five times with 300 μL of EIA buffer and 100 μL of streptavidin conjugated with HRP were added. After 1 h incubation and a washing step, 100 μL of TMB substrate were added to each well and the plates were incubated for about 30–60 min at RT. The reaction was terminated with a 100 μL/well of 2 N HCl. Absorbance was read at λ = 450 nm on Infinite 200 (Tecan).

The ELISA four parameter curve was prepared for each neuropeptide (an Excel sheet was provided by Penisula Laboratories service). Samples were assayed in duplicate. The concentration in each sample was read from the curve. Final peptide concentration was presented as ng per g of analyzed tissue and presented as the mean from group ± SD.

### Statistical analyses

The results were analyzed statistically using a one-way analysis of variance (ANOVA) and the significance of differences between groups was determined using Duncan’s multiple-range test at a significance level of p < 0.05. The data are expressed as mean values ± SD. Calculations were made with SigmaPlot^®^ 12 (Systat Software Inc.)
